# Clinical-Metabolic Risk Phenotypes and Carotid Plaque Burden Among Community-Dwelling Individuals at High Cardiovascular Risk: An Exploratory Cross-Sectional Study

**DOI:** 10.3390/jcdd13090409

**Published:** 2026-08-24

**Authors:** Zechang Lv, Dazhi Wang, Yubin Chen, Ziheng Xiao, Rui Lu, Benrong Liu, Wenchao Ou

**Affiliations:** Department of Cardiology, Guangzhou Institute of Cardiovascular Disease, Guangdong Key Laboratory of Vascular Diseases, State Key Laboratory of Respiratory Disease, the Second Affiliated Hospital, Guangzhou Medical University, Guangzhou 510260, China; lvzechang0810@163.com (Z.L.); m13213731148@163.com (D.W.); chenyub2000@163.com (Y.C.); 19050348454@163.com (Z.X.); 17358850038@163.com (R.L.)

**Keywords:** high cardiovascular risk, clinical-metabolic phenotype, carotid plaque burden, carotid ultrasound, subclinical atherosclerosis, clustering analysis, community-based study

## Abstract

Background: Community-dwelling individuals at high cardiovascular risk are clinically heterogeneous, but whether distinct clinical-metabolic phenotypes differ in carotid plaque burden remains unclear. Methods: From 4191 participants in a community-based screening project conducted in two communities in Guangzhou, 1015 individuals in the high-cardiovascular-risk stage with nonmissing high-plaque-burden status were included. All 1015 participants underwent exploratory clustering using 17 clinical, metabolic, lifestyle-related, and disease-history variables. High plaque burden was defined as bilateral carotid plaques, multiple plaques, or maximum plaque thickness at or above the 75th percentile among plaque-positive participants. Logistic regression examined associations between phenotypes and high plaque burden. Results: Among the 1015 participants included in the clustering analysis, 329 (32.4%) met the definition of high plaque burden. Four phenotypes were identified: relatively low metabolic burden, older age–ASCVD burden, smoking–low HDL-C, and blood pressure-loaded phenotypes. The corresponding prevalences of high plaque burden were 15.9%, 44.4%, 49.7%, and 33.2%. After adjustment for age and sex, all three non-reference phenotypes showed higher odds of high plaque burden. In sensitivity analysis excluding age and prior ASCVD from clustering, the blood pressure-loaded phenotype remained associated with high plaque burden. Conclusions: Four exploratory clinical-metabolic phenotypes showed different levels of carotid plaque burden among community-dwelling individuals at high cardiovascular risk. The blood pressure-loaded and smoking–low HDL-C phenotypes were associated with greater plaque burden and may provide an exploratory framework for describing heterogeneity within high-risk populations.

## 1. Introduction

Community-based cardiovascular screening is an important strategy for identifying individuals who may benefit from preventive management before the occurrence of major cardiovascular events [1,2]. In such settings, individuals classified as being at high cardiovascular risk represent a priority population for further evaluation, risk-factor control, and vascular health assessment [1,3]. This issue is particularly relevant in China, where cardiovascular disease burden and hypertension prevalence remain substantial [4,5]. However, the conventional label of “high cardiovascular risk” is broad and may conceal substantial clinical heterogeneity within this population [6].

In practice, individuals who enter a high-risk stage may have markedly different clinical-metabolic profiles. One person may be characterized predominantly by elevated blood pressure, another by clustered smoking and low high-density lipoprotein cholesterol (HDL-C) and another by older age and prior atherosclerotic cardiovascular disease (ASCVD). These patterns reflect the fact that cardiovascular risk factors rarely occur in isolation [7,8]. Instead, major atherosclerotic vascular risk factors, including age, blood pressure, lipid profiles, glucose metabolism, adiposity, and smoking behavior, may coexist in different combinations [7,8]. In individuals who have entered a high-risk stage, disease-history variables such as prior ASCVD may further contribute to phenotypic heterogeneity. Lipid abnormalities, diabetes, and elevated blood glucose have been consistently associated with vascular outcomes [9,10]. Metabolic syndrome frameworks also emphasize the clustering of adiposity, blood pressure, and lipid and glucose abnormalities [11,12]. A phenotype-based description may therefore help move beyond a single high-risk label and better characterize the heterogeneity of community-dwelling high-risk individuals.

Carotid ultrasound provides a noninvasive assessment of subclinical atherosclerotic burden [13,14]. Among individuals already considered to be at high cardiovascular risk, carotid plaque burden may capture clinically relevant differences in vascular status that are not fully reflected by routine risk classification alone [15,16]. Two individuals with the same high-risk label may differ considerably in the extent of underlying carotid atherosclerosis [17,18]. Prior studies have also linked carotid intima-media thickness, plaque presence, and ultrasound plaque features to cardiovascular risk profiles [19,20]. Contemporary community and high-risk cohort studies further support the relevance of carotid ultrasound markers in vascular risk assessment [21,22]. Population-based evidence has also linked carotid plaques and hypertension with cardiovascular outcomes [23]. Therefore, examining carotid plaque burden across different clinical-metabolic profiles may provide additional insight into vascular heterogeneity within high-risk populations.

Existing analyses of cardiovascular risk in community populations often focus on individual risk factors, predefined disease categories, or overall risk classification [1,2]. Although these approaches are clinically useful, they may not fully describe how multiple risk features coexist within the same individual or whether different combinations of risk features correspond to different levels of subclinical atherosclerotic burden. Data-driven clustering analysis offers a way to characterize naturally occurring patterns of clinical and metabolic features and to provide a phenotype-based view of within-population heterogeneity [24,25].

The present study aimed to identify clinical-metabolic risk phenotypes among community-dwelling individuals at high cardiovascular risk and to examine whether carotid plaque burden differed across these phenotypes. By characterizing heterogeneity within a community-based high-risk population, this study may provide a basis for refining the description of cardiovascular risk profiles and generating hypotheses for phenotype-informed prevention research.

## 2. Materials and Methods

### 2.1. Study Design and Population

The community-based cardiovascular screening project was conducted in 2025 among permanent residents of the Nanshitou and Zhuguang communities in Guangzhou, China. Eligible residents were invited through community notice boards and resident WeChat groups, and participation was voluntary. Eligibility required birth between 1 January 1950 and 31 December 1990, residence in the relevant community for at least 6 months during the preceding 12 months, and written informed consent. Initial screening was conducted from March to June 2025, followed by imaging examinations among high-risk participants from June to September 2025.

The original screening included 4191 participants. After excluding 84 participants with missing key examination data, 4107 participants had complete core data. According to the high-risk-stage assessment records in the original screening program, 1054 participants were classified as being in the high-cardiovascular-risk stage, whereas 3053 were classified as non-high-risk individuals. The present analysis was restricted to the 1054 participants in the high-cardiovascular-risk stage.

**Figure 1:**
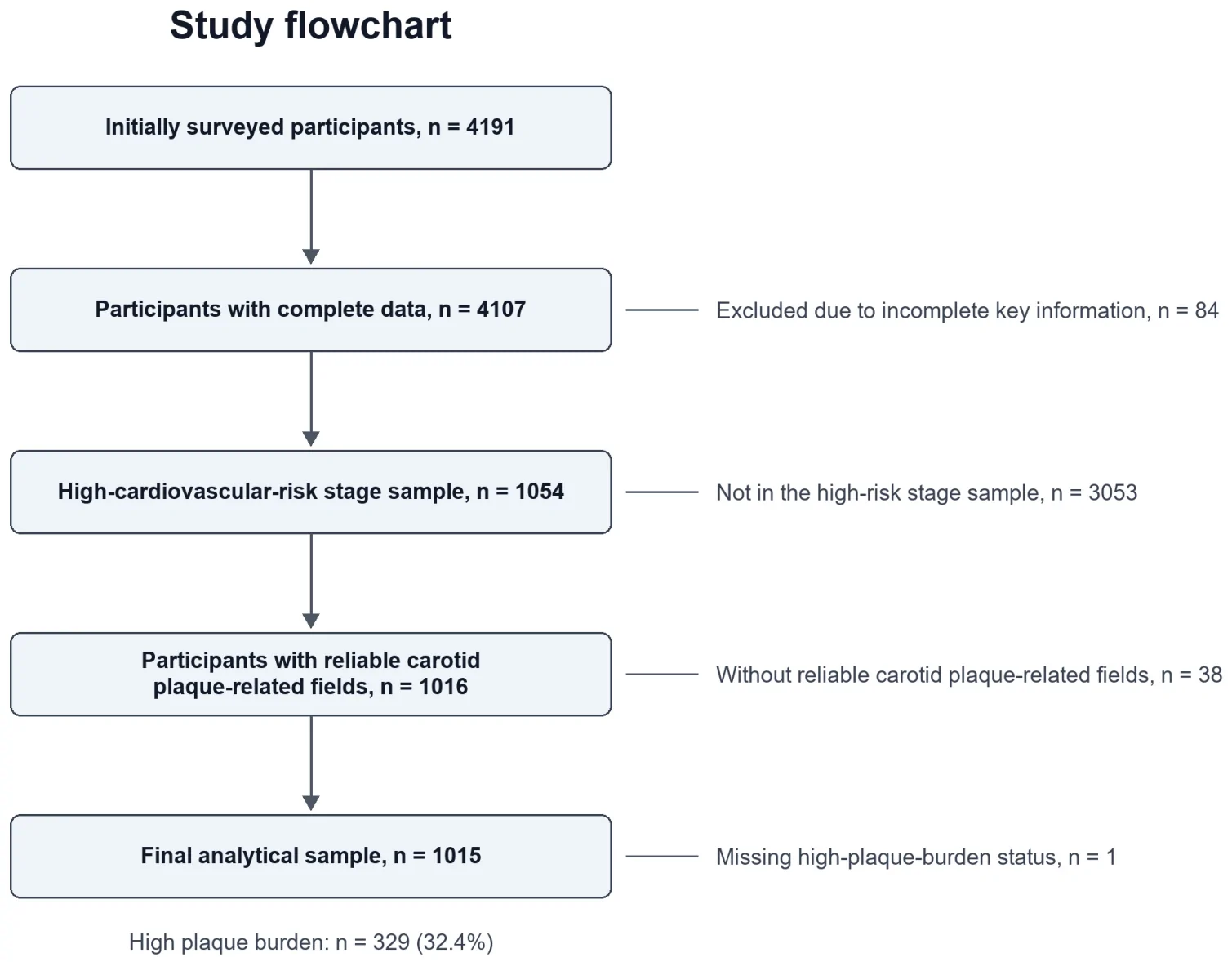
Study flowchart.

Among these high-risk participants, 1016 had reliable carotid plaque-related fields, and 1015 had nonmissing high plaque burden status. All 1015 participants were included in the exploratory clustering analysis, whereas the 329 participants with high plaque burden represented the outcome events. The same final analytic sample was used for descriptive comparisons and regression analyses. The participant selection process is shown in Figure 1.

The study was reported with reference to the Strengthening the Reporting of Observational Studies in Epidemiology (STROBE) statement [26].

### 2.2. Definition of High-Cardiovascular-Risk Stage

High-cardiovascular-risk status was determined according to the high-risk-stage assessment records of the original community screening program. The high-risk-stage assessment was based on established criteria, including prior atherosclerotic cardiovascular disease (ASCVD), markedly elevated blood pressure, extreme lipid abnormality, or an estimated 10-year ASCVD risk of 10% or higher [1,6].

Prior ASCVD included myocardial infarction, percutaneous coronary intervention, coronary artery bypass grafting, ischemic stroke, and hemorrhagic stroke. Participants with recorded prior ischemic or hemorrhagic stroke were not excluded. Markedly elevated blood pressure was defined as mean systolic blood pressure ≥ 160 mmHg or mean diastolic blood pressure ≥ 100 mmHg. Extreme lipid abnormality was defined as low-density lipoprotein cholesterol (LDL-C) ≥ 4.14 mmol/L or high-density lipoprotein cholesterol (HDL-C) ≤ 0.78 mmol/L.

In the present analysis, the high-cardiovascular-risk stage sample was identified according to the original high-risk-stage assessment records, which reflected entry into the high-risk assessment module of the screening program.

### 2.3. Carotid Ultrasound Assessment

Following the initial screening, all participants classified as being at high cardiovascular risk underwent carotid ultrasonography between June and September 2025. Examinations were performed by qualified sonographers according to standardized operating procedures using a high-frequency linear-array transducer. The bilateral common carotid arteries, carotid bifurcations, and proximal internal carotid arteries were examined.

Carotid intima-media thickness was measured at the distal 1 cm segment of the common carotid artery. A carotid plaque was defined as a focal intima-media thickness of at least 1.5 mm or a focal thickening of at least 50% relative to the surrounding vessel wall. Plaque counts for the left and right carotid arteries and maximum plaque thickness were obtained from the original ultrasound records. Total plaque count was calculated as the sum of the recorded left and right plaque counts.

### 2.4. Definition of High Plaque Burden

The primary endpoint was high plaque burden, which was defined using plaque laterality, plaque number, and maximum plaque thickness [13,17]. Participants were classified as having high plaque burden if they met at least one of the following criteria: bilateral carotid plaques, multiple carotid plaques, or increased maximum plaque thickness.

Bilateral carotid plaques were defined as a left carotid plaque count greater than 0 and a right carotid plaque count greater than 0. Multiple carotid plaques were defined as the sum of left and right carotid plaque counts greater than or equal to 2. Increased maximum plaque thickness was defined as maximum plaque thickness at or above the 75th percentile among plaque-positive participants (2.5 mm).

### 2.5. Variables Used for Exploratory Clustering Analysis

The exploratory clustering analysis used 17 demographic, clinical, metabolic, lifestyle-related, and disease-history variables: age, body mass index, waist circumference, mean systolic blood pressure, mean diastolic blood pressure, blood glucose, total cholesterol, triglycerides, LDL-C, HDL-C, sleep duration, sex, smoking status, history of hypertension, history of diabetes, history of dyslipidemia, and prior ASCVD.

Mean systolic and diastolic blood pressure were derived from screening blood pressure measurements. Histories of hypertension, diabetes, dyslipidemia, and ASCVD were obtained from the original screening records based on self-reported physician diagnosis and/or documented medical history.

No carotid ultrasound-derived variables were included in the clustering procedure. Therefore, carotid plaque burden was not used to define the clinical-metabolic risk phenotypes and was evaluated only after phenotype assignment.

### 2.6. Exploratory Clustering Analysis

Continuous variables were checked using clinically plausible ranges before analysis. Continuous variables were standardized before clustering to reduce the influence of scale differences. Missing continuous variables were imputed using the median, and missing binary variables were imputed using the mode. Near-constant binary variables were not included in the clustering procedure.

K-means clustering was used to identify exploratory clinical-metabolic risk phenotypes [27]. The groups were not defined according to a pre-existing clinical classification. The four-cluster solution was retained as an exploratory phenotype framework based on clinical interpretability, adequate subgroup size, and avoidance of very small or poorly interpretable clusters.

The four clinical-metabolic risk phenotypes were labeled according to their dominant standardized features: relatively low metabolic burden phenotype, older age–ASCVD burden phenotype, smoking–low HDL-C phenotype, and blood pressure-loaded phenotype. These labels were assigned descriptively after examination of the dominant standardized cluster features and were not intended to represent validated clinical classifications.

### 2.7. Statistical Analysis

Baseline characteristics and carotid plaque burden indicators were summarized across the four clinical-metabolic risk phenotypes. Categorical variables were presented as counts and percentages, and continuous variables were presented as means and standard deviations or medians and interquartile ranges, as appropriate.

Logistic regression was used to estimate odds ratios (ORs) and 95% confidence intervals (CIs) for the association between clinical-metabolic risk phenotypes and high plaque burden. The relatively low-metabolic-burden phenotype served as the reference group. The primary regression model adjusted for age and sex.

A robustness model further adjusted for smoking status, body mass index, waist circumference, systolic blood pressure, diastolic blood pressure, blood glucose, LDL-C, HDL-C, triglycerides, history of hypertension, history of diabetes, history of dyslipidemia, and prior ASCVD. Because these covariates substantially overlapped with variables used to define the clusters, this model was interpreted cautiously and was considered a robustness analysis rather than the primary inferential model.

Cluster stability was assessed using bootstrap resampling and the adjusted Rand index [28,29]. Sensitivity analyses were performed by excluding age and prior ASCVD from the clustering procedure. In this sensitivity framework, an automatically selected two-cluster solution and a fixed four-cluster solution were examined. Logistic regression was then used to evaluate the association between the sensitivity-derived phenotypes and high plaque burden. To examine the role of sex in the smoking–low HDL-C phenotype, a phenotype-by-sex interaction was tested in the logistic regression analysis. The four-cluster analysis was also repeated after excluding sex, and agreement with the original cluster assignments was assessed using the adjusted Rand index. For descriptive visualization, participant-level pairwise scatter plots and a principal-component projection were used to illustrate cluster separation and overlap. Principal components were interpreted according to the variables with the largest absolute loadings.

All analyses were interpreted as associations. A two-sided *p* value <0.05 was considered statistically significant.

### 2.8. Ethical Considerations

This study was conducted in accordance with the Declaration of Helsinki and approved by the Clinical Research and Application Ethics Committee of The Second Affiliated Hospital of Guangzhou Medical University (Approval No. KY-EC2025-001-01; date of approval: 11 April 2025). Informed consent was obtained from all subjects involved in the study.

## 3. Results

### 3.1. Study Population and High Plaque Burden

As shown in Figure 1, 1015 community-dwelling individuals at high cardiovascular risk were included in the final analysis and clustering procedure. Among them, 329 participants met the definition of high plaque burden, corresponding to a prevalence of 32.4%.

### 3.2. Clinical-Metabolic Risk Phenotypes

**Figure 2:**
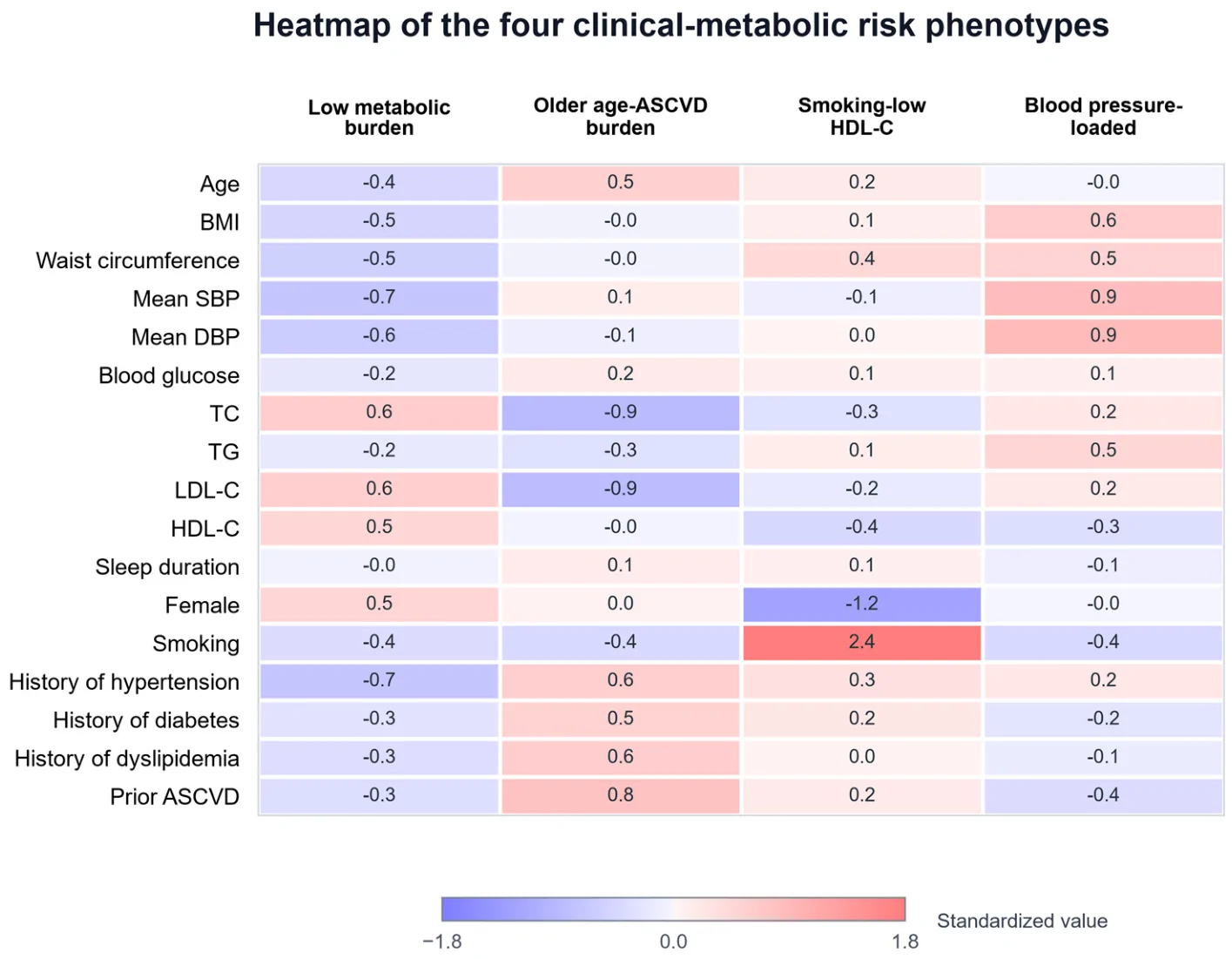
Heatmap of the four clinical-metabolic risk phenotypes. Standardized values are shown for clustering variables. ASCVD, atherosclerotic cardiovascular disease; BMI, body mass index; DBP, diastolic blood pressure; HDL-C, high-density lipoprotein cholesterol; LDL-C, low-density lipoprotein cholesterol; SBP, systolic blood pressure; TC, total cholesterol; TG, triglycerides.

Exploratory clustering analysis identified four clinical-metabolic risk phenotypes. The relatively low metabolic burden phenotype included 347 participants (34.2%) and was characterized by comparatively lower blood pressure, adiposity measures, blood glucose, and prevalences of major disease histories. However, total cholesterol and LDL-C levels were higher in this phenotype; therefore, the label refers to the dominant overall pattern within this high-risk population rather than uniformly favorable metabolic characteristics or low absolute cardiovascular risk. The older age–ASCVD burden phenotype included 252 participants (24.8%) and was characterized by older age and a higher burden of prior ASCVD and cardiometabolic disease history. The smoking–low HDL-C phenotype included 145 participants (14.3%) and was characterized predominantly by smoking and lower HDL-C. The blood pressure-loaded phenotype included 271 participants (26.7%) and was characterized by the highest systolic and diastolic blood pressure, together with higher adiposity measures. The clinical-metabolic input profiles of the four exploratory clusters are summarized in Table 1, and their standardized feature patterns are shown in Figure 2. Participant-level scatter plots and principal-component visualization showed partial separation together with overlap among the four clusters (Supplementary Figure S1).

### 3.3. Carotid Plaque Burden Across Phenotypes

**Figure 3:**
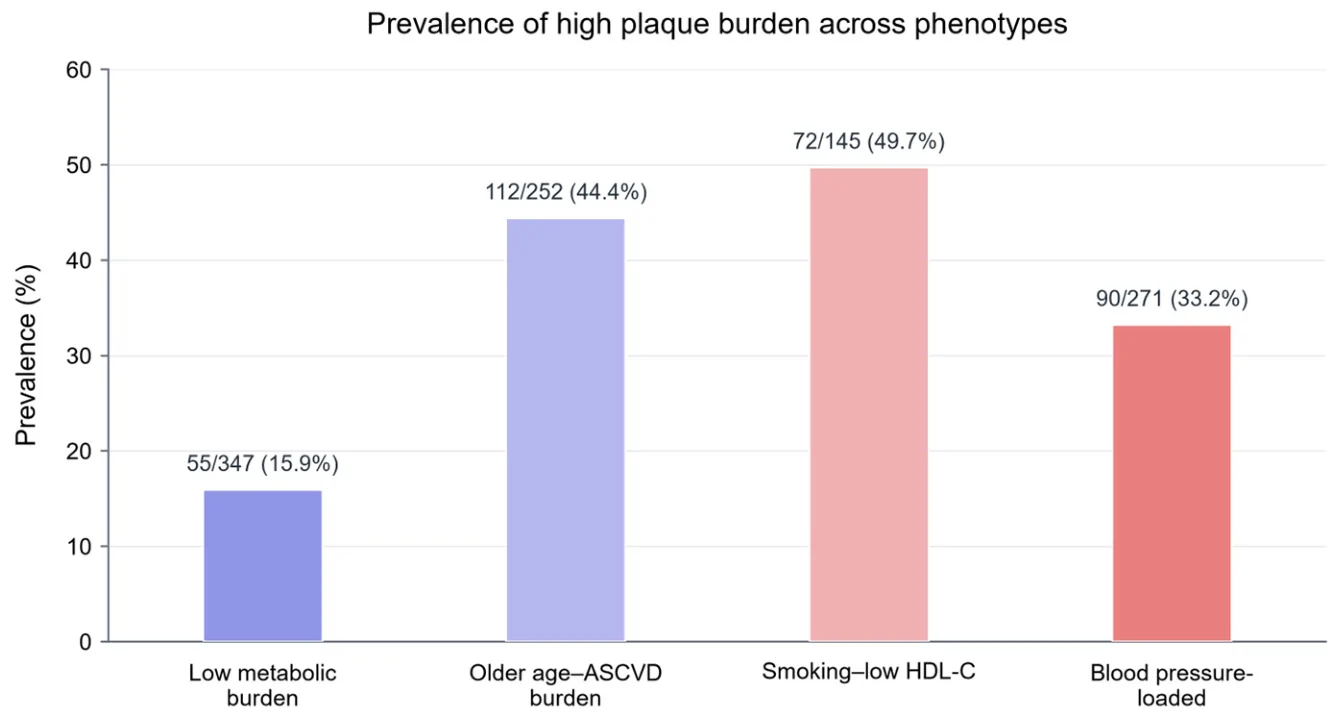
Prevalence of high plaque burden across clinical-metabolic risk phenotypes. High plaque burden was defined as bilateral carotid plaques, multiple plaques, or maximum plaque thickness at or above the 75th percentile among plaque-positive participants.

Carotid plaque burden differed across the four clinical-metabolic risk phenotypes. The prevalence of high plaque burden was lowest in the relatively low metabolic burden phenotype and highest in the smoking–low HDL-C phenotype. Specifically, high plaque burden was present in 15.9% of participants in the relatively low metabolic burden phenotype, 44.4% in the older age–ASCVD burden phenotype, 49.7% in the smoking–low HDL-C phenotype, and 33.2% in the blood pressure-loaded phenotype. Among plaque-positive participants, the overall median total plaque count was 2 (IQR, 1–3), and the median maximum plaque thickness was 2.0 mm (IQR, 1.6–2.5 mm). The smoking–low HDL-C phenotype had the highest median maximum plaque thickness at 2.1 mm (IQR, 1.7–2.8 mm), whereas the relatively low metabolic burden phenotype had the lowest median total plaque count at 1 (IQR, 1–2). Carotid plaque burden indicators across phenotypes are summarized in Table 2, and the prevalence of high plaque burden is shown in Figure 3.

### 3.4. Association Between Clinical-Metabolic Risk Phenotypes and High Plaque Burden

**Figure 4:**
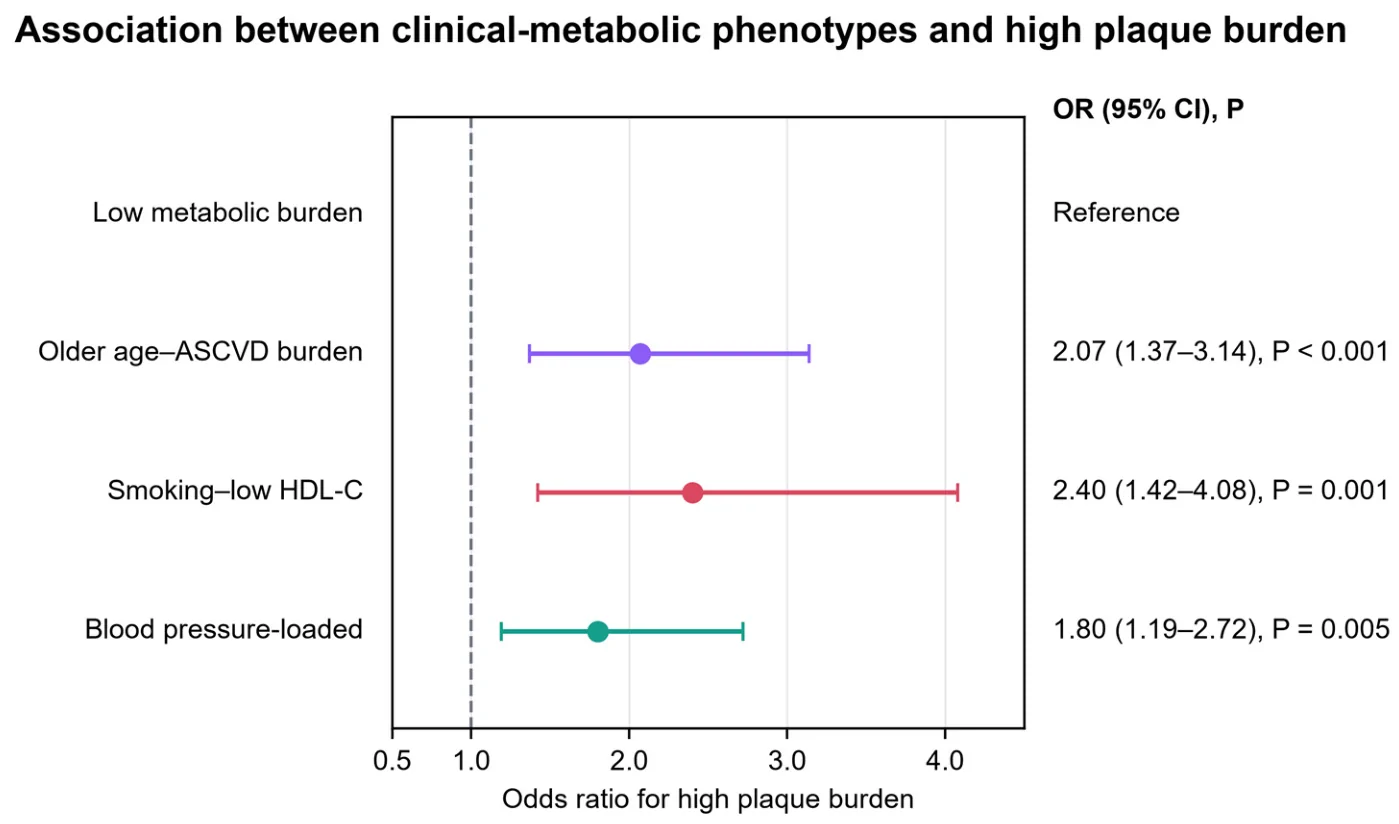
Association between clinical-metabolic phenotypes and high plaque burden. Odds ratios were adjusted for age and sex. The relatively low-metabolic-burden phenotype was used as the reference group.

After adjustment for age and sex, all three non-reference phenotypes showed higher odds of high plaque burden compared with the relatively low-metabolic-burden phenotype. The adjusted ORs were 2.07 for the older age–ASCVD burden phenotype (95% CI 1.37–3.14, *p* < 0.001), 2.40 for the smoking–low HDL-C phenotype (95% CI 1.42–4.08, *p* = 0.001), and 1.80 for the blood pressure-loaded phenotype (95% CI 1.19–2.72, *p* = 0.005). The logistic regression results are shown in Table 3 and Figure 4.

### 3.5. Cluster Stability and Sensitivity Analyses

The original four-cluster solution showed moderate stability, with a bootstrap mean adjusted Rand index (ARI) of 0.536 and a median ARI of 0.504. After excluding age and prior ASCVD from the clustering procedure, the automatically selected two-cluster solution identified a dyslipidemia phenotype and a blood pressure-loaded phenotype. Across both sensitivity solutions, the dyslipidemia phenotype was characterized primarily by higher TC and LDL-C, whereas HDL-C was not consistently low and TG and recorded dyslipidemia history were not consistently elevated. The prevalence of high plaque burden was 19.8% in the dyslipidemia phenotype and 44.3% in the blood pressure-loaded phenotype. After adjustment for age and sex, the blood pressure-loaded phenotype remained associated with higher odds of high plaque burden compared with the dyslipidemia phenotype (OR 2.10, 95% CI 1.53–2.87, *p* < 0.001).

**Figure 5:**
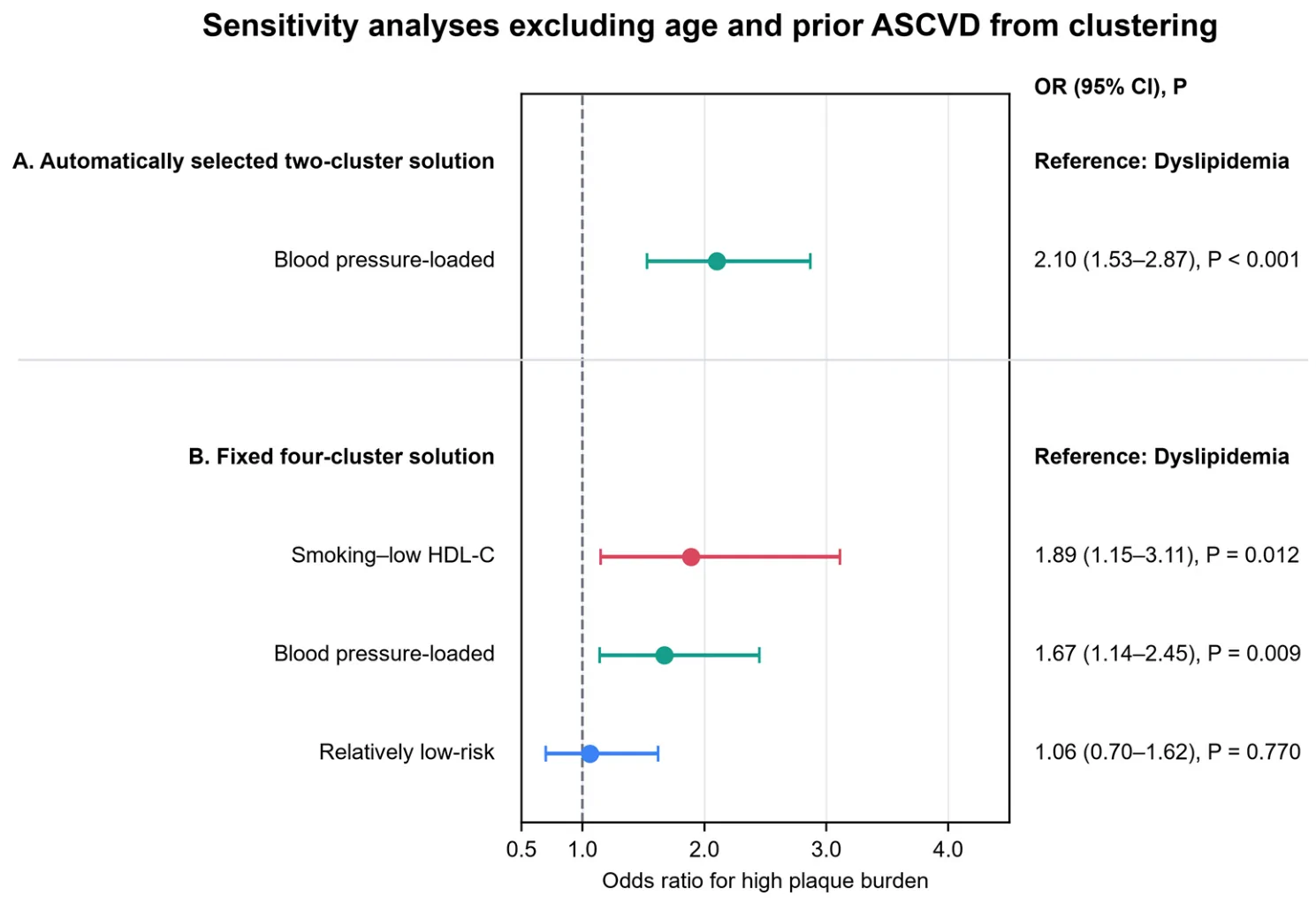
Sensitivity analyses excluding age and prior ASCVD from clustering. Panel (**A**) shows the automatically selected two-cluster solution, and Panel (**B**) shows the fixed four-cluster solution. Odds ratios were adjusted for age and sex, with the dyslipidemia phenotype as the reference group.

In the fixed four-cluster sensitivity analysis excluding age and prior ASCVD, the prevalence of high plaque burden was 22.0% in the dyslipidemia phenotype, 24.5% in the relatively low-risk phenotype, 49.7% in the smoking–low HDL-C phenotype, and 41.6% in the blood pressure-loaded phenotype. Compared with the dyslipidemia phenotype, the smoking–low HDL-C phenotype (OR 1.89, 95% CI 1.15–3.11, *p* = 0.012) and the blood pressure-loaded phenotype (OR 1.67, 95% CI 1.14–2.45, *p* = 0.009) showed higher odds of high plaque burden, whereas the relatively low-risk phenotype did not (OR 1.06, 95% CI 0.70–1.62, *p* = 0.770). The sensitivity analyses excluding age and prior ASCVD are summarized in Table 4 and Figure 5. The standardized cluster profiles of the two sensitivity solutions are shown in Supplementary Figure S2. In additional analyses examining the role of sex, the phenotype-by-sex interaction for high plaque burden was not statistically significant (*p* = 0.092). When the four-cluster analysis was repeated without sex, agreement with the original cluster assignments was moderate (adjusted Rand index = 0.444), suggesting that sex contributed to, but did not solely determine, the observed cluster structure.

## 4. Discussion

This community-based cross-sectional study identified four clinically interpretable clinical-metabolic phenotypes among individuals at high cardiovascular risk and showed that carotid plaque burden varied substantially across these phenotypes. Rather than representing a homogeneous group, high-cardiovascular-risk individuals encompassed distinct patterns of risk-factor aggregation, including relatively low metabolic burden, older age with prior ASCVD burden, smoking–low HDL-C, and blood pressure loading. These findings suggest that a single high-risk label may not fully capture the heterogeneity of vascular burden within community-dwelling high-risk populations [6,24].

The older age–ASCVD burden phenotype had a higher carotid plaque burden, which was consistent with the accumulation of vascular disease markers among older individuals and those with established ASCVD history [16,30]. This phenotype likely reflects a subgroup in whom chronological aging and prior clinical vascular disease coexist with a greater burden of subclinical carotid atherosclerosis. Although unsurprising, this finding emphasizes that vascular burden remains highly heterogeneous even within an already high cardiovascular-risk population.

The blood pressure-loaded phenotype was characterized by the highest measured systolic and diastolic blood pressure, although it did not have the highest prevalence of self-reported hypertension history. This discrepancy may reflect previously unrecognized hypertension, suboptimal blood pressure control, or transient blood pressure elevation during screening. However, these possibilities could not be confirmed because antihypertensive medication information and repeated out-of-office blood pressure measurements were unavailable. The persistence of a blood pressure-loaded pattern after excluding age and prior ASCVD from the clustering procedure suggests that elevated measured blood pressure remained an important dimension of heterogeneity within this high-risk population [31,32].

The smoking–low HDL-C phenotype also showed a high prevalence of carotid plaque burden. This phenotype should not be interpreted as a simple adiposity phenotype. Instead, it appeared to represent a clustered lifestyle-metabolic risk pattern characterized by prominent smoking and lower HDL-C [7,33]. The coexistence of these features may help explain why this phenotype showed a high burden of carotid plaque despite not being defined by older age or prior ASCVD [34,35]. Men accounted for 142 of the 145 participants in this phenotype. The phenotype-by-sex interaction for high plaque burden was not statistically significant (*p* = 0.092), and the four-cluster analysis excluding sex showed moderate agreement with the original solution (adjusted Rand index = 0.444), suggesting that sex contributed to, but did not solely determine, the phenotype structure. Because smoking was self-reported and the phenotype was strongly male-predominant, the findings should be interpreted with caution and evaluated in external datasets.

The moderate bootstrap stability of the original four-cluster solution is also worth noting [28,29]. This level of stability is compatible with the exploratory nature of the analysis and supports the use of the four-cluster solution as a phenotype framework for describing heterogeneity within this study population [24,29]. At the same time, the sensitivity analysis excluding age and prior ASCVD showed that the original phenotype structure was partly shaped by age and established vascular disease history. The persistence of the blood pressure-loaded axis in this sensitivity analysis strengthens the interpretation that blood pressure burden is an important dimension of clinical-metabolic heterogeneity in this population.

These findings may have implications for community-based cardiovascular prevention. The identified phenotypes may help highlight different dominant risk-factor patterns within an already high-risk population. For example, the blood pressure-loaded phenotype may prompt careful assessment of blood pressure control, whereas the smoking–low HDL-C phenotype may reinforce the importance of smoking cessation and lipid management. The older age–ASCVD burden phenotype may warrant particular attention to established vascular disease and secondary prevention. However, current management should continue to follow established guideline-based assessment and treatment, and the additional clinical value of phenotype-based characterization requires prospective validation. Accordingly, the present phenotypes should be regarded primarily as an exploratory descriptive framework, with limited immediate clinical applicability.

This study has several strengths. First, it focused on a community-based population already classified as being at high cardiovascular risk, a group that is highly relevant to preventive cardiovascular care. Second, the clustering procedure used routinely available clinical, metabolic, lifestyle-related, and disease-history variables, which enhances the interpretability of the identified phenotypes. Third, carotid plaque burden was evaluated after phenotype assignment and was not used to define the clusters, reducing circularity between phenotype construction and outcome assessment. Finally, the analysis included bootstrap stability assessment and sensitivity analyses excluding age and prior ASCVD, which helped evaluate the robustness of the observed phenotype patterns.

Several limitations should be acknowledged. First, the cross-sectional design and lack of prospective cardiovascular outcome data precluded assessment of temporal relationships, causal inference, or the ability of the identified phenotypes to predict future ASCVD events [26]. Second, the study was based on community screening data from a limited regional setting, which may limit generalizability to other populations. Third, plaque area and volume were unavailable in the screening dataset; therefore, indicators based on plaque laterality, number, and maximum thickness may not fully capture total geometric plaque burden. Fourth, carotid stenosis was not analyzed because the available project documentation did not provide a standardized Doppler-velocity protocol or a validated stenosis-grading algorithm. Fifth, the clustering analysis was exploratory and data-driven; therefore, the identified phenotypes should not be interpreted as formal clinical classification standards. Sixth, although bootstrap analysis supported the four-cluster solution as an exploratory phenotype framework, external validation is still required. Seventh, some lifestyle-related variables were questionnaire-derived and may be subject to recall or reporting bias.

## 5. Conclusions

In this community-based high-cardiovascular-risk population, four exploratory clinical-metabolic phenotypes were identified and showed different levels of carotid plaque burden. The blood pressure-loaded and smoking–low HDL-C phenotypes were associated with greater plaque burden, and the blood pressure-loaded phenotype remained evident after excluding age and prior ASCVD from clustering. These findings provide an exploratory framework for describing vascular heterogeneity within high-risk populations and may inform future phenotype-based research.

## Figures and Tables

**Table 1:** Clinical-metabolic input profiles of the four exploratory clusters.

Characteristic	Relatively Low-Metabolic-Burden Phenotype	Older Age–ASCVD Burden Phenotype	Smoking–Low HDL-C Phenotype	Blood Pressure-Loaded Phenotype
n (%)	347 (34.2%)	252 (24.8%)	145 (14.3%)	271 (26.7%)
Age	58.26 ± 8.91	66.69 ± 5.88	63.68 ± 7.73	62.05 ± 8.53
Female	292 (84.1%)	160 (63.5%)	3 (2.1%)	165 (60.9%)
BMI	22.37 ± 2.95	23.93 ± 3.11	24.39 ± 3.30	26.12 ± 3.62
Waist circumference	79.19 ± 8.27	84.24 ± 9.45	88.77 ± 9.51	89.43 ± 8.64
Mean SBP	118.25 ± 14.72	135.57 ± 17.72	131.13 ± 16.97	151.47 ± 18.77
Mean DBP	73.31 ± 7.59	79.28 ± 8.90	80.28 ± 9.81	89.89 ± 10.24
Blood glucose	6.08 ± 1.20	6.77 ± 1.75	6.60 ± 2.10	6.59 ± 1.95
TC	6.72 ± 1.26	4.50 ± 0.87	5.37 ± 1.52	6.12 ± 1.25
TG	1.74 ± 0.81	1.60 ± 0.69	2.08 ± 1.29	2.46 ± 1.44
LDL-C	4.05 ± 1.18	2.05 ± 0.73	3.00 ± 1.32	3.52 ± 1.24
HDL-C	1.97 ± 0.53	1.72 ± 0.49	1.50 ± 0.52	1.55 ± 0.43
Sleep duration	7.88 ± 1.19	8.12 ± 1.26	8.05 ± 1.57	7.80 ± 1.18
Smoking	6 (1.7%)	1 (0.4%)	145 (100.0%)	1 (0.4%)
History of hypertension	31 (8.9%)	182 (72.2%)	85 (58.6%)	146 (53.9%)
History of diabetes	31 (8.9%)	102 (40.5%)	42 (29.0%)	28 (10.3%)
History of dyslipidemia	70 (20.2%)	164 (65.1%)	55 (37.9%)	82 (30.3%)
Prior ASCVD	9 (2.6%)	102 (40.5%)	29 (20.0%)	3 (1.1%)

The variables presented in this table were included in the clustering procedure and are shown descriptively to characterize the composition of each cluster; they should not be interpreted as independent associations with cluster membership. Values are shown as mean ± standard deviation or n (%). Abbreviations: ASCVD, atherosclerotic cardiovascular disease; BMI, body mass index; HDL-C, high-density lipoprotein cholesterol; LDL-C, low-density lipoprotein cholesterol; SBP, systolic blood pressure; DBP, diastolic blood pressure; TC, total cholesterol; TG, triglycerides.

**Table 2:** Carotid plaque burden indicators across the four clinical-metabolic risk phenotypes.

Carotid Plaque Burden Indicator	Overall	Relatively Low-Metabolic-Burden Phenotype	Older Age–ASCVD Burden Phenotype	Smoking–Low HDL-C Phenotype	Blood Pressure-Loaded Phenotype
Carotid plaque, n/N (%)	523/1015 (51.5%)	111/347 (32.0%)	166/252 (65.9%)	99/145 (68.3%)	147/271 (54.2%)
Total plaque count, median (IQR)	1 (0–2)	0 (0–1)	1 (0–2)	1 (0–3)	1 (0–2)
Total plaque count among plaque-positive participants, median (IQR)	2 (1–3)	1 (1–2)	2 (1–3)	2 (1–4)	2 (1–3)
Bilateral plaque, n/N (%)	265/1015 (26.1%)	44/347 (12.7%)	94/252 (37.3%)	59/145 (40.7%)	68/271 (25.1%)
Multiple plaques, n/N (%)	308/1015 (30.3%)	50/347 (14.4%)	106/252 (42.1%)	67/145 (46.2%)	85/271 (31.4%)
Maximum plaque thickness among plaque-positive participants, mm, median (IQR)	2.0 (1.6–2.5)	1.9 (1.6–2.1)	2.0 (1.7–2.6)	2.1 (1.7–2.8)	1.9 (1.6–2.5)
Maximum plaque thickness ≥ P75, n/N (%)	141/1015 (13.9%)	19/347 (5.5%)	46/252 (18.3%)	34/145 (23.4%)	42/271 (15.5%)
High plaque burden, n/N (%)	329/1015 (32.4%)	55/347 (15.9%)	112/252 (44.4%)	72/145 (49.7%)	90/271 (33.2%)

Values are presented as event count/denominator (%) or median (interquartile range). P75 indicates the 75th percentile of maximum plaque thickness among plaque-positive participants and corresponds to 2.5 mm.

**Table 3:** Logistic regression analysis of clinical-metabolic phenotypes and high plaque burden.

Model	Reference	Phenotype	OR (95% CI)	*p*
Crude	Relatively low-metabolic-burden phenotype	Blood pressure-loaded phenotype	2.64 (1.80–3.87)	<0.001
Crude	Relatively low-metabolic-burden phenotype	Older age–ASCVD burden phenotype	4.25 (2.90–6.21)	<0.001
Crude	Relatively low-metabolic-burden phenotype	Smoking–low HDL-C phenotype	5.24 (3.39–8.09)	<0.001
Age-sex adjusted	Relatively low-metabolic-burden phenotype	Blood pressure-loaded phenotype	1.80 (1.19–2.72)	0.005
Age-sex adjusted	Relatively low-metabolic-burden phenotype	Older age–ASCVD burden phenotype	2.07 (1.37–3.14)	<0.001
Age-sex adjusted	Relatively low-metabolic-burden phenotype	Smoking–low HDL-C phenotype	2.40 (1.42–4.08)	0.001
Fully adjusted exploratory	Relatively low-metabolic-burden phenotype	Blood pressure-loaded phenotype	1.66 (0.91–3.02)	0.098
Fully adjusted exploratory	Relatively low-metabolic-burden phenotype	Older age–ASCVD burden phenotype	1.21 (0.65–2.25)	0.540
Fully adjusted exploratory	Relatively low-metabolic-burden phenotype	Smoking–low HDL-C phenotype	3.47 (0.32–37.16)	0.303

The relatively low-metabolic-burden phenotype was used as the reference group. OR, odds ratio; CI, confidence interval. The fully adjusted model was exploratory because the adjusted covariates substantially overlapped with the variables used to define the clusters.

**Table 4:** Sensitivity analyses excluding age and prior ASCVD from clustering.

Analysis	Phenotype or Comparison	High Plaque Burden, n/N (%)	Estimate	*p*
Original four-cluster solution	Bootstrap stability	329/1015 (32.4%)	Mean ARI = 0.536; median ARI = 0.504	—
Automatic two-cluster solution excluding age and prior ASCVD	Dyslipidemia phenotype	98/494 (19.8%)	Reference	—
Automatic two-cluster solution excluding age and prior ASCVD	Blood pressure-loaded phenotype	231/521 (44.3%)	OR 2.10 (1.53–2.87)	<0.001
Fixed four-cluster solution excluding age and prior ASCVD	Dyslipidemia phenotype	67/305 (22.0%)	Reference	—
Fixed four-cluster solution excluding age and prior ASCVD	Relatively low-risk phenotype	65/265 (24.5%)	OR 1.06 (0.70–1.62)	0.770
Fixed four-cluster solution excluding age and prior ASCVD	Smoking–low HDL-C phenotype	73/147 (49.7%)	OR 1.89 (1.15–3.11)	0.012
Fixed four-cluster solution excluding age and prior ASCVD	Blood pressure-loaded phenotype	124/298 (41.6%)	OR 1.67 (1.14–2.45)	0.009
Stability excluding age and prior ASCVD	Automatic two-cluster solution	—	Mean ARI = 0.864; median ARI = 0.863	—
Stability excluding age and prior ASCVD	Fixed four-cluster solution	—	Mean ARI = 0.551; median ARI = 0.493	—

ARI, adjusted Rand index; ASCVD, atherosclerotic cardiovascular disease; OR, odds ratio; CI, confidence interval. Logistic regression models were adjusted for age and sex. In each sensitivity regression analysis, the dyslipidemia phenotype was used as the reference group.

## Data Availability

The data supporting the findings of this study are not publicly available due to privacy and ethical restrictions. De-identified data may be made available from the corresponding author upon reasonable request and with appropriate ethical approval.

## Supplementary Materials

The following supporting information can be downloaded at https://www.mdpi.com/article/10.3390/jcdd13090409/s1. Supplementary Figure S1. Participant-level visualization of the four exploratory clinical-metabolic risk phenotypes. Panels A–C show BMI versus waist circumference, mean systolic versus diastolic blood pressure, and HDL-C versus waist circumference, respectively. Panel D presents scores for the first two principal components derived from the 17 standardized clustering variables; PC1 and PC2 explained 18.8% and 13.4% of the total variance, respectively. PC1 received its largest absolute contributions from history of hypertension, TC, waist circumference, LDL-C, and mean SBP, whereas PC2 received its largest absolute contributions from TC, LDL-C, BMI, waist circumference, and TG. Small circles represent individual participants, and X symbols indicate phenotype centroids. Colors denote the four-cluster assignments. Partial overlap is expected because the phenotypes were identified from the joint multivariable profile rather than from any single variable or two-dimensional boundary. ASCVD, atherosclerotic cardiovascular disease; BMI, body mass index; DBP, diastolic blood pressure; HDL-C, high-density lipoprotein cholesterol; LDL-C, low-density lipoprotein cholesterol; PC, principal component; SBP, systolic blood pressure; TC, total cholesterol; TG, triglycerides. Supplementary Figure S2. Heatmaps of standardized cluster centroids after excluding age and prior ASCVD from the clustering variables. Panel A shows the automatically selected two-cluster solution, and Panel B shows the fixed four-cluster solution. Values represent mean z-scores for the remaining 15 clustering variables. Both panels use the same color scale. ASCVD, atherosclerotic cardiovascular disease; DBP, diastolic blood pressure; HDL-C, high-density lipoprotein cholesterol; LDL-C, low-density lipoprotein cholesterol; SBP, systolic blood pressure; TC, total cholesterol; TG, triglycerides.

## Abbreviations

The following abbreviations are used in this manuscript:

ARI	Adjusted Rand index
ASCVD	Atherosclerotic cardiovascular disease
BMI	Body mass index
CI	Confidence interval
DBP	Diastolic blood pressure
HDL-C	High-density lipoprotein cholesterol
LDL-C	Low-density lipoprotein cholesterol
OR	Odds ratio
SBP	Systolic blood pressure
TC	Total cholesterol
TG	Triglycerides
