# Supplementary material for: Clinical-Metabolic Risk Phenotypes and Carotid Plaque Burden Among Community-Dwelling Individuals at High Cardiovascular Risk: An Exploratory Cross-Sectional Study
Source: J Cardiovasc Dev Dis. 2026 Aug 24;13(9):409. doi: 10.3390/jcdd13090409 (PMC13607766; doi:10.3390/jcdd13090409)
Supplement: Supplementary file 1 [file jcdd-13-00409-s001.zip › jcdd-4457181-supplementary.pdf]

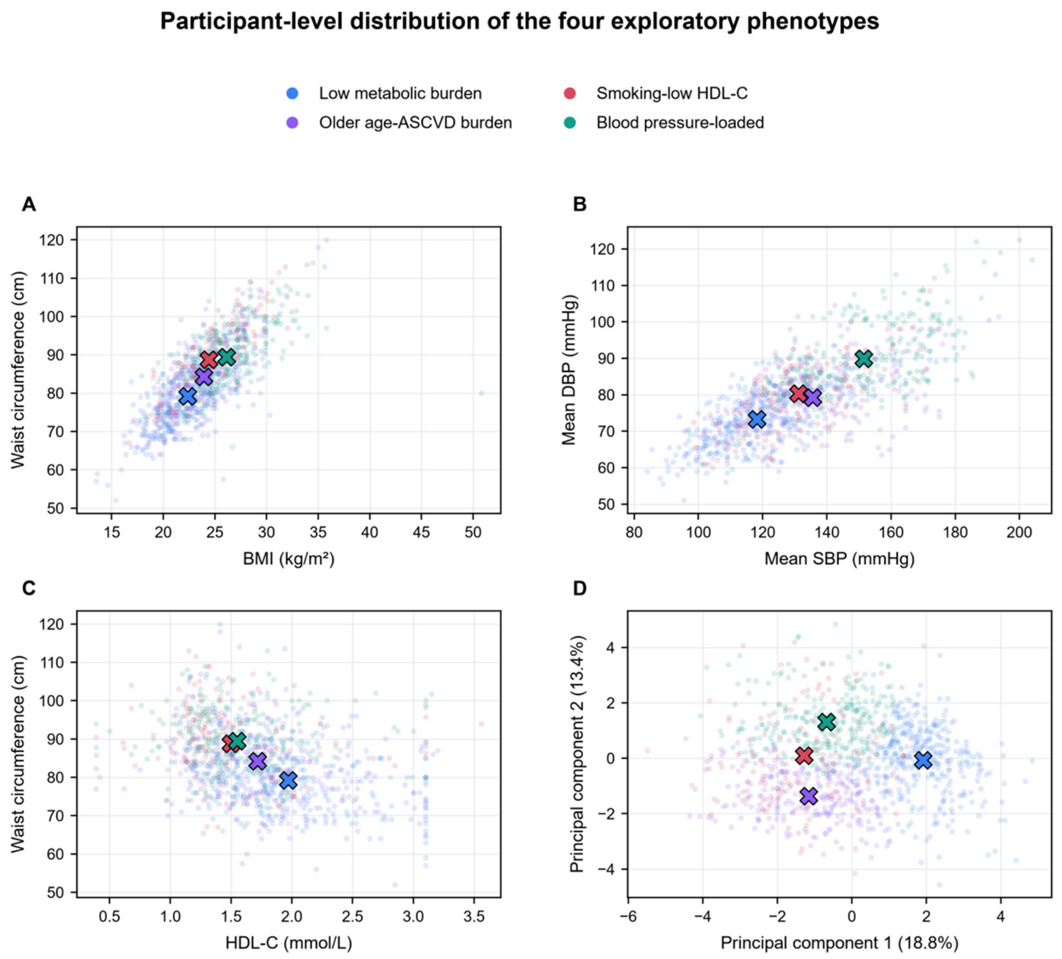

Supplementary Figure S1. Participant-level visualization of the four exploratory clinical-metabolic risk phenotypes. Panels A–C show BMI versus waist circumference, mean systolic versus diastolic blood pressure, and HDL-C versus waist circumference, respectively. Panel D presents scores for the first two principal components derived from the 17 standardized clustering variables; PC1 and PC2 explained 18.8% and 13.4% of the total variance, respectively. PC1 received its largest absolute contributions from history of hypertension, TC, waist circumference, LDL-C, and mean SBP, whereas PC2 received its largest absolute contributions from TC, LDL-C, BMI, waist circumference, and TG. Small circles represent individual participants, and X symbols indicate phenotype centroids. Colors denote the four-cluster assignments. Partial overlap is expected because the phenotypes were identified from the joint multivariable profile rather than from any single variable or two-dimensional

boundary. ASCVD, atherosclerotic cardiovascular disease; BMI, body mass index; DBP, diastolic blood pressure; HDL-C, high-density lipoprotein cholesterol; LDL-C, low-density lipoprotein cholesterol; PC, principal component; SBP, systolic blood pressure; TC, total cholesterol; TG, triglycerides.

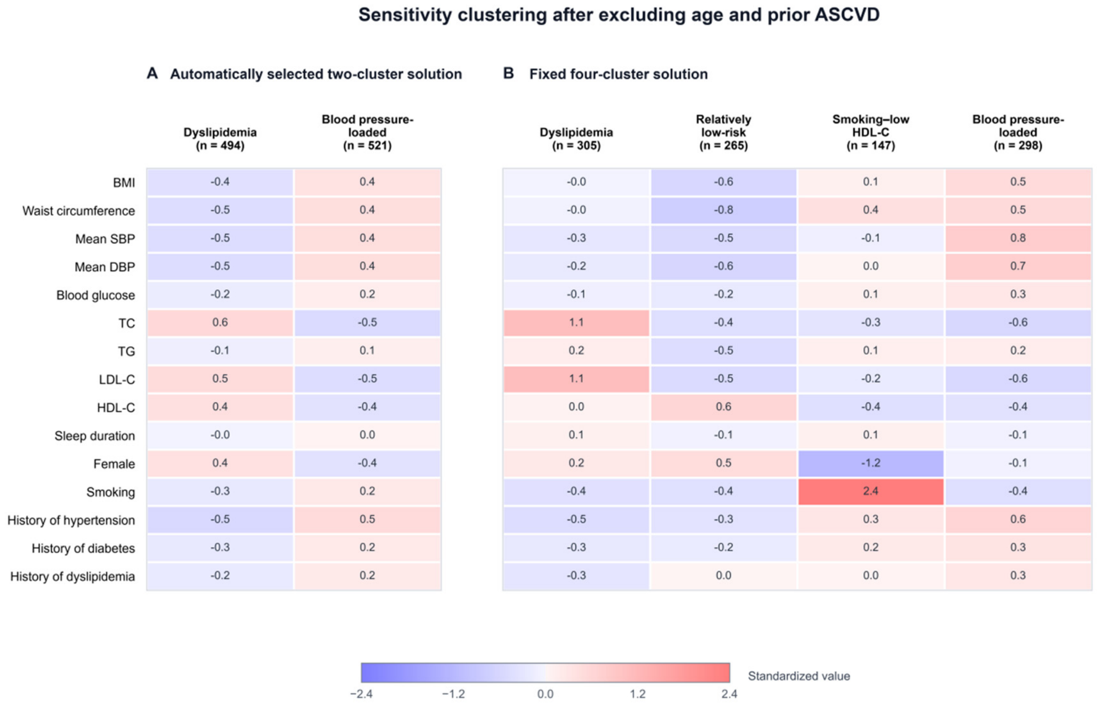

	A: Dyslipidemia (n = 494)	A: Blood pressure-loaded (n = 521)	B: Dyslipidemia (n = 305)	B: Relatively low-risk (n = 265)	B: Smoking–low HDL-C (n = 147)	B: Blood pressure-loaded (n = 298)
BMI	-0.4	0.4	-0.0	-0.6	0.1	0.5
Waist circumference	-0.5	0.4	-0.0	-0.8	0.4	0.5
Mean SBP	-0.5	0.4	-0.3	-0.5	-0.1	0.8
Mean DBP	-0.5	0.4	-0.2	-0.6	0.0	0.7
Blood glucose	-0.2	0.2	-0.1	-0.2	0.1	0.3
TC	0.6	-0.5	1.1	-0.4	-0.3	-0.6
TG	-0.1	0.1	0.2	-0.5	0.1	0.2
LDL-C	0.5	-0.5	1.1	-0.5	-0.2	-0.6
HDL-C	0.4	-0.4	0.0	0.6	-0.4	-0.4
Sleep duration	-0.0	0.0	0.1	-0.1	0.1	-0.1
Female	0.4	-0.4	0.2	0.5	-1.2	-0.1
Smoking	-0.3	0.2	-0.4	-0.4	2.4	-0.4
History of hypertension	-0.5	0.5	-0.5	-0.3	0.3	0.6
History of diabetes	-0.3	0.2	-0.3	-0.2	0.2	0.3
History of dyslipidemia	-0.2	0.2	-0.3	0.0	0.0	0.3

Supplementary Figure S2. Heatmaps of standardized cluster centroids after excluding age and prior ASCVD from the clustering variables. Panel A shows the automatically selected two-cluster solution, and Panel B shows the fixed four-cluster solution. Values represent mean z-scores for the remaining 15 clustering variables. Both panels use the same color scale. ASCVD, atherosclerotic cardiovascular disease; DBP, diastolic blood pressure; HDL-C, high-density lipoprotein cholesterol; LDL-C, low-density lipoprotein cholesterol; SBP, systolic blood pressure; TC, total cholesterol; TG, triglycerides.
